# Feasibility of intensity-modulated and image-guided radiotherapy for locally advanced esophageal cancer

**DOI:** 10.1186/1471-2407-14-265

**Published:** 2014-04-17

**Authors:** Nam P Nguyen, Siyoung Jang, Jacqueline Vock, Vincent Vinh-Hung, Alexander Chi, Paul Vos, Judith Pugh, Richard A Vo, Misty Ceizyk, Anand Desai, Lexie Smith-Raymond

**Affiliations:** 1 Department of Radiation Oncology, Howard University Hospital, 2401 Georgia Avenue, N.W., Room 2055, Washington, DC 20060, USA; 2 Department of Radiation Oncology, Lindenhofspital, Bern, Switzerland; 3 Department of Radiation Oncology, University Hospitals of Geneva, Geneva, Switzerland; 4 Department of Radiation Oncology, University of West Virginia, Morgantown, WV, USA; 5 Department of Biostatistics, East Carolina University, Greenville, NC, USA; 6 Department of Pathology, University of Arizona, Tucson, AZ, USA; 7 Department of Pediatry, University of Virginia, Charlottesville, VA, USA; 8 Department of Radiation Oncology, Akron City Hospital, Akron, OH, USA; 9 Department of Radiation Oncology, University of Arizona, Tucson, AZ, USA

**Keywords:** Esophageal cancer, Tomotherapy, Normal tissue sparing

## Abstract

**Background:**

In this study the feasibility of intensity-modulated radiotherapy (IMRT) and tomotherapy-based image-guided radiotherapy (IGRT) for locally advanced esophageal cancer was assessed.

**Methods:**

A retrospective study of ten patients with locally advanced esophageal cancer who underwent concurrent chemotherapy with IMRT (1) and IGRT (9) was conducted. The gross tumor volume was treated to a median dose of 70 Gy (62.4-75 Gy).

**Results:**

At a median follow-up of 14 months (1-39 months), three patients developed local failures, six patients developed distant metastases, and complications occurred in two patients (1 tracheoesophageal fistula, 1 esophageal stricture requiring repeated dilatations). No patients developed grade 3-4 pneumonitis or cardiac complications.

**Conclusions:**

IMRT and IGRT may be effective for the treatment of locally advanced esophageal cancer with acceptable complications.

## Background

Treatment of locally advanced esophageal cancer remains a significant challenge because of the high rate of loco-regional and distant failures [1]. Preoperative chemoradiation is usually advocated for better loco-regional control in selected patients with adequate cardio-pulmonary reserve. However, morbidity following surgery remains high with a 46% rate of pulmonary and a 21% rate of cardiac complications [2]. For inoperable patients, standard of care has been concurrent chemoradiation [3,4]. Radiation dose was usually limited to 50 Gy in the U.S. because of the increased toxicity associated with a higher dose without survival improvement [3]. However, recent studies demonstrated that high radiation dose for esophageal cancer may be feasible and in selected studies provided similar survival compared to surgery [5-7]. Current radiotherapy techniques are limited by the radiation dose that can be safely delivered to the gross tumor without increasing the risk of pneumonitis and cardiac toxicity. Thus, a radiotherapy technique that reduces treatment toxicity while providing a curative dose of radiation to the tumor may improve survival and local control. Intensity-modulated radiotherapy (IMRT) has been introduced to improve target coverage while potentially decreasing radiation dose to the normal tissues [8-10]. Compared to three-dimensional conformal radiotherapy (3D-CRT), IMRT significantly reduced radiation dose to the heart and coronary arteries for distal esophageal cancer [10]. The myocardium sparing effect of IMRT may explain why esophageal cancer patients treated with IMRT had less cardiac complications and better survival compared to the ones treated with 3D-CRT [11]. A new technique of IMRT delivery, helical tomotherapy based image-guided radiotherapy (IGRT) provides steeper dose gradient and target coverage compared to conventional IMRT for patients with esophageal cancer [12]. In a previous dosimetric comparison study, we also demonstrated that tomotherapy provided better sparing of the heart and lungs compared to 3D-CRT for distal esophageal cancers [13]. In the current study, we report the clinical outcome of patients with esophageal cancers treated with IMRT and IGRT to assess whether these radiotherapy techniques may also be effective for loco-regional control with acceptable toxicity.

## Methods

The medical records of 10 patients undergoing radiotherapy for esophageal cancer at the University of Arizona Radiation Oncology department were retrospectively identified. The University of Arizona Institutional Board (IRB) approved the study. Prior to radiotherapy treatment, all patients signed informed consent for radiotherapy treatment, and also agreed for publication of the data, including imaging following de-identification in the consent form. Patients were selected if they had esophageal cancer treated with IMRT (1) or IGRT (9) for possible cure. The IGRT was performed with the Tomotherapy HD Unit with daily pretreatment CT imaging. All patients received concurrent definitive chemoradiation (8) or postoperative chemoradiation for positive margins (2). Patients selected for definitive chemoradiation were not candidates for resection because of multiple co-morbidities. Prior to treatment, each patient was simulated in the supine position with a body vacuum bag for treatment immobilization. A computed tomography (CT) scan with and without oral and intravenous (IV) contrast for treatment planning was performed in the treatment position. The chest and upper abdomen were scanned with a slice thickness of 3 mm. The CT scan with oral and IV contrast was employed to outline the tumor and grossly enlarged regional lymph node for target volume delineation. Radiotherapy planning was performed on the CT scan without contrast to avoid possible interference of contrast density on radiotherapy isodose distributions. Diagnostic positron emission tomography (PET)-CT scan planning for tumor imaging was also incorporated with CT planning when available. Normal organs at risk for complication were outlined for treatment planning (spinal cord, cardiac ventricles, lungs, kidney, liver, and bowels). The cardiac ventricles (right and left) were contoured on the contrast CT scan. The gross tumor volume (GTV) was outlined by integrating information obtained from the CT scan with IV and oral contrast study and PET-CT scan when available. Clinical target volume (CTV) was expanded with a 0.3-0.5 cm radial expansion and a 5-cm superior-inferior expansion. The celiac lymph nodes were also included in the CTV for patients with cancer of the distal esophagus cancer or the gastro-esophageal junctions with an expansion of 0.5 cm. Any mediastinal lymph nodes enlargement observed on CT scan and/or PET scan were also included in the CTV. The PTV was defined as 0.5 cm beyond the CTV. The integrated boost technique was used for both techniques to treat the PTV to 45 Gy at 1.8 Gy/fraction and the GTV to 50 Gy at 2 Gy/fraction respectively. One patient had hypofractionation to the GTV at 2.2 Gray/fraction (37.4 Gy) as the PTV as treated to 30.6 Gy at 1.8 Gy/fraction. The patient had a very large tumor and we had to limit the total dose delivered because of lungs constraint. Dose constraints for normal organs at risk (OAR) for complications were: spinal cord (Dmax <45 Gy), total lung (V5 < 50%, V10 < 40%, V15 < 30%, and V20 < 25%), cardiac ventricles (V10 < 50%), liver (V30 < 30%), kidneys (V15 < 30%), and bowels (V45 < 50%). A minimum of 95% coverage was required for both tomotherapy and IMRT plans. At 40 Gy to the GTV or 37.4 Gy for the patient treated with hypofractionation, a CT scan with oral and intravenous contrast was repeated with the patient in the treatment position to assess tumor shrinkage with radiation. The residual gross tumor was boosted with a one cm margin to a achieve a gross total tumor dose of 20 Gy in 2 Gy/fraction or 25 Gy in 1.25 Gy twice a day (bid) for patients with definitive chemoradiation to bring the total gross tumor dose to 70 Gy and 75 Gy respectively. The patient who had hypofractionation received a GTV boost of 25 Gy in 2.5 Gy/fraction to compensate for the lower tumor dose in the previous treatment plan. The cumulative gross tumor dose for that patient was 62.4 Gy. The bid boost fractionation was selected in patients with tumors adherent to the vessels or trachea to decrease the risk of complications and in postoperative patients with positive margins to reduce the risks of anastomotic leaks. Among the two patients with positive margins, the former GTV was boosted with the hyperfractionation schedule (1.25 Gy bid) to 15 Gy (cumulative gross tumor dose 65 Gy) and 20 Gy (cumulative gross tumor dose 70 Gy). The second patient had a higher tumor boost dose (20 Gy) because of the delay in initiating radiotherapy after surgery.

All patients underwent concurrent chemotherapy with 5-fluorouracil (5-FU) at 1000 mg/m^2^/24 hours by continuous intravenous (IV) infusion on days 1-4, and cisplatin (cisp) at 100 mg/m^2^ IV bolus on day 1 of radiotherapy. Chemoradiotherapy was repeated on day 28 of radiotherapy. Prophylactic percutaneous endoscopic gastrostomy (PEG) feeding-tube placement was also recommended for all patients because of the expected esophagitis and weight loss during treatment. Weekly complete blood count (CBC) and blood chemistry work-ups were performed during chemoradiation. Treatment breaks and weight loss were recorded during chemoradiation. Acute and long-term toxicities were graded according to Radiotherapy Oncology Group (RTOG) group criteria severity scales (http://www.rtog.org./). Acute toxicity was monitored during the course of treatment. Long-term toxicity was recorded at each patient follow-up visit.

The patients were evaluated one month after treatment, every three months for two years, then every six months for two years, then yearly. A PET-CT scan was repeated four months, ten months, then yearly following treatment. An endoscopic exam was repeated three to four months after treatment, then yearly, unless there was suspicion of local recurrences on PET-CT or clinically (dysphagia recurrence, gastrointestinal bleeding). A repeated biopsy was performed if there was suspicion of recurrences on endoscopic exam. All patients were monitored closely by a team of dietitians during, and following treatment to assess their nutritional status and tube feedings. Survival analysis was performed using Kaplan-Meier estimation.

## Results

We identified 10 patients with locally advanced esophageal cancer (3 T3, 7 T4) treated at the University of Arizona Radiation Oncology department from 2008 to 2010. Median age at diagnosis was 58 years (range: 49-75 years). There were eight males and two females. The histology was squamous (**n = 3**) and adenocarcinoma (**n = 7**). The tumor was located in the upper third (**n = 1**), mid third (**n = 2**) and lower third (**n = 7**). Eight patients had definitive chemoradiation and two patients had postoperative chemoradiation. Table 1 summarizes patients characteristics.

**Table 1:** Patient characteristics

Patient number:		10
Age:		
	Range	49-75
	Median	58
Sex:		
	Male	8
	Female	2
Tumor location		
	Upper third	1
	Middle third	2
	Lower third	7
Histology		
	Squamous	3
	Adenocarcinoma	7
Tumor stage		
	T3	3
	T4	7
Nodal stage		
	N0	2
	N1	5
	N2	3
Treatment		
	IMRT	1
	IGRT	9
	Definitive chemoradiation	8
	Postoperative chemoradiation	2
Gross tumor dose (Gy)		
	62.4	1
	65	1
	70	5
	75	3
Boost technique		
	Hypofractionation (2.5 Gy)	1
	Hyperfractionation (1.25 Gy bid)	4
	Conventional fractionation (2 Gy)	5
Follow-up (months)		
	Range	1-38
	Median	14

Gy: gray; bid: twice a day.

At a median follow-up of 14 months (range: 1-38 months), local recurrences developed in three patients. All three recurrences occurred in the area of GTV receiving high dose of radiation within four, six, and eleven months after radiation. Six patients developed distant metastases (bones: 3, lungs: 1, liver: 1, abdomen and pelvis: 2). Two patients developed second lung primaries. One patient died from his second primary and the other one was salvaged with stereotactic body radiotherapy. No patient developed regional lymph nodes recurrences. The causes of death were local recurrence (1), distant metastases (4), second primary (1), and pneumonia (1). The 2-year and 3-year survival is estimated to be 50% and 40% for the whole group.

Two patients developed grade 3-4 toxicity during treatment (1 esophagitis and neutropenia, 1 pneumonia). Chemotherapy was discontinued in one patient after the first cycle because of pneumonia and one patient had chemotherapy delayed after the first cycle because of grade 4 neutropenia. All patients completed their radiotherapy treatment. Only three patients had treatment breaks ranging from 4 to 14 days. Median weight loss was 4 kilograms (0-10 kilograms).

At a median follow-up of 14 months, two patient developed complications. One patient had esophageal stricture requiring repeated dilatations. The other patient developed tracheoesophageal fistula requiring placement of a stent. She eventually died from liver metastases.

**Figure 1:**
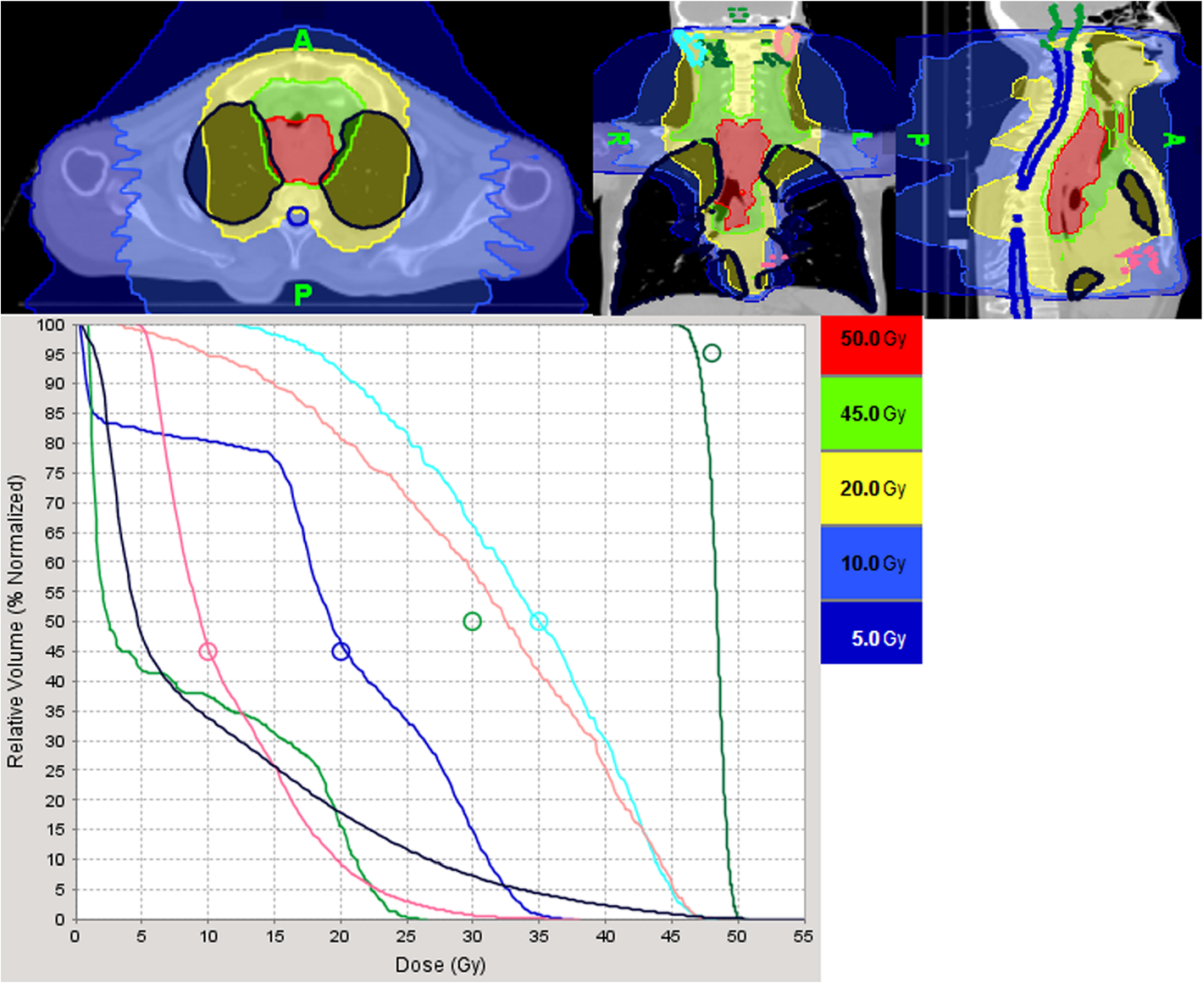
**Illustration of the dose-volume histogram of tomotherapy planning for a patient with a T4N1M0 squamous cell carcinoma extending from the cricoid cartilage to the lower third of the esophagus.** The cervical, mediastinal, and celiac lymph nodes were treated to 45 Gy in 180 Gy/fraction while the gross tumor was treated with an integrated boost technique to 50 Gy in 2 Gy/fraction. The black line illustrates the total lung dose. Despite the large tumor size, the volume of lungs treated to 20 Gy (V20), 15 Gy (V15), 10 Gy (V10) and 5 Gy (V5) was 18%, 25%, 38%, and 50%, respectively. Maximum spinal cord dose and brain stem dose was 37 Gy (dark blue line) and 27 Gy (light green), respectively. The pink line illustrates the radiation dose to the cardiac ventricles. The blue and light maroon line illustrates the dose to the right and left parotid respectively.

**Figure 2:**
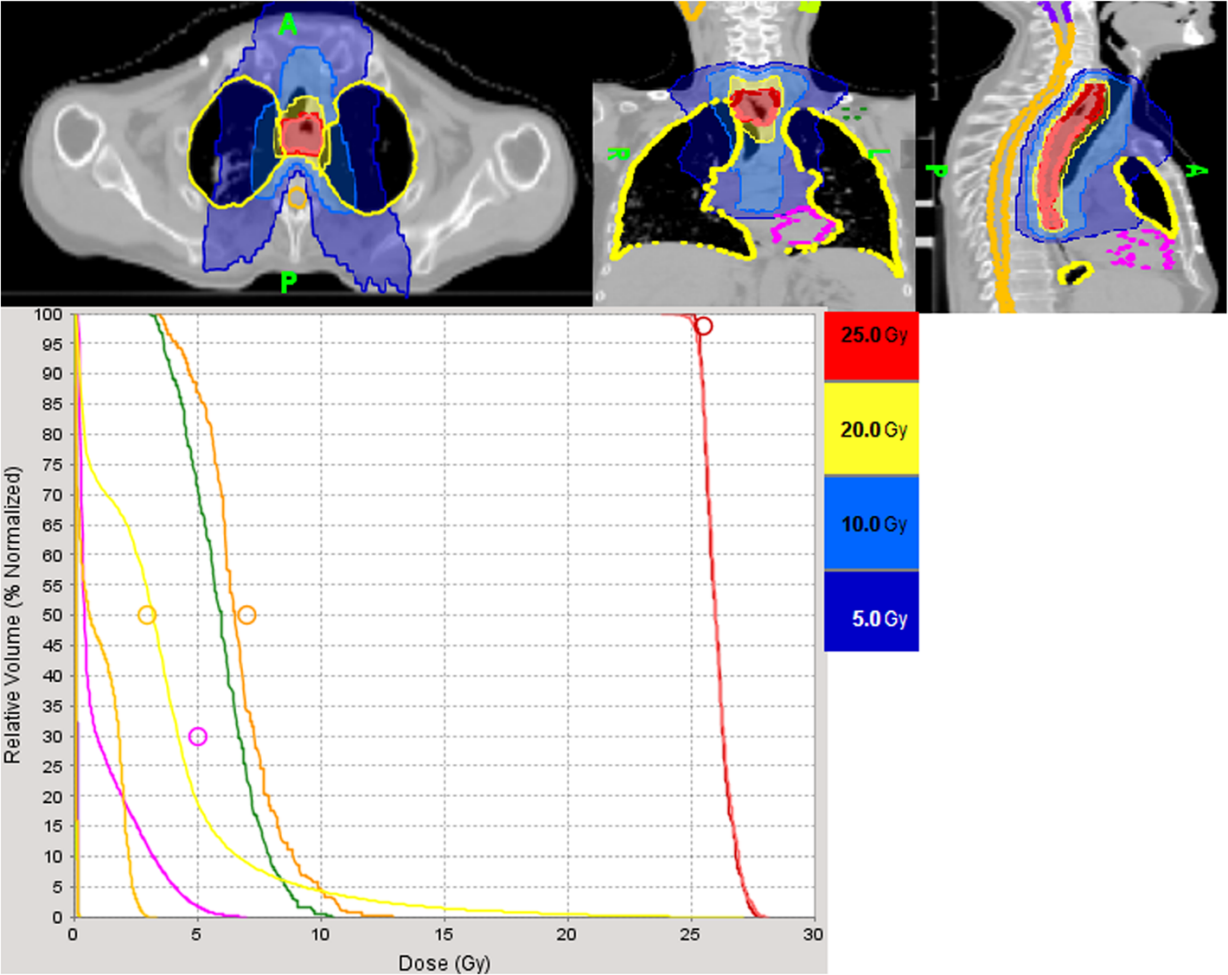
**Illustration of the dose-volume histogram of the tumor boost in the same patient on the planning CT repeated at 40 Gy.** The gross tumor had decreased in size significantly and was boosted to 25 Gy at 1.25 Gy twice a day to decrease the risk of complications because of the tumor close proximity to the trachea and blood vessels. The yellow line and pink line illustrates the dose to the lungs and cardiac ventricles. Maximum spinal cord dose was 3 Gy (orange). The brown and dark line illustrates the dose to the right and left brachial plexus.

Figures 1 and 2 illustrate the feasibility of tomotherapy to deliver a high dose of radiation to the gross tumor volume while sparing the normal heart and lungs. The patient had a circumferential squamous cell carcinoma extending from the thoracic esophagus to the cervical and distal esophagus and invading the mediastinal lymph nodes (T4N1M0) on PET-CT scan. The patient also had congestive heart failure and severe chronic obstructive pulmonary disease fromexcessive smoking and binge drinking. He was cachectic because the tumor produced complete obstruction of the esophagus requiring emergency placement of a PEG tube for feeding. The gross tumor and regional lymph nodes (cervical, mediastinal, and celiac lymph nodes) were treated to 50 Gy and 45 Gy respectively with the integrated boost technique). A repeated planning CT scan at 40 Gy demonstrated significantreduction of the gross tumor which was then boosted to 25 Gy in 1.25 Gy bid because of the close proximity of the tumor to the trachea. The patient had a complete response to the treatment on repeated endoscopic exam and sequential PET-CT scans. He was able to resume oral feedings and gained weight after treatment but required multiple dilatations because of esophageal stenosis secondary to scarring. The patient continued to smoke despite multiple medical advices against smoking and developed a poorly differentiated T1N0M0 carcinoma of the left upper lung lobe thought to be a second primary three years after radiation. He is now undergoing stereotactic body radiotherapy for salvage as he is not a candidate for lobectomy because of his medical condition.

## Discussion

To our knowledge, our study confirms the feasibility of IGRT to deliver a high dose of radiation with acceptable complications in patients who were unable to undergo resection because of associated co-morbidities (8) or who had positive margins following surgery (2). Chen et al. [14] also reported the feasibility of IGRT in 10 patients with locally advanced esophageal cancers who were unable to undergo surgery because of disease extent and/or associated co-morbidities. However, the gross tumor volume was only treated to 50 Gy with the integrated boost technique similar to our study. All patients in our study had a repeated planning CT scan during treatment to assess tumor shrinkage and the residual tumor was boosted to improve local control. Among the patients who had definitive chemoradiation, only one out of eight patients had no tumor shrinkage during treatment. Using this technique, we were able to deliver a curative dose of radiation with a conventional fractionation (5), hyperfractionation (4) or hypofractionation (1) schedule. Even though the patient number is small and the follow-up short, only three patients recurred locally which is encouraging because most of the patients had locally advanced disease (7 patients T4). All local recurrences occurred in the GTV area receiving a high dose of radiation emphasizing the fact that a radiation dose of 66-70 Gy may not be adequate for tumor control. As an illustration, Bedenne et al. [5] reported a local recurrence rate of 43% despite a radiation dose up to 66 Gy to the GTV in patients with esophageal cancer undergoing definitive chemoradiation. In another randomized study for patients with T3-T4 esophageal cancer undergoing chemoradiation to a total dose up to 65 Gy, loco-regional control was achieved in only 30% of the patients [6]. The simultaneous integrated boost (SIB) technique allows delivery of a high dose of radiation to the gross tumor volume and sparing of radiosensitive organs such as the rectum in patients with prostate cancer [15]. The adaptive radiotherapy technique by taking advantage of the tumor shrinkage may spare the adjacent normal tissues while still delivering a high radiation dose to the PTV [16,17]. As most of the recurrences following definitive chemoradiation occur within the gross tumor volume and are associated with T3-T4 lesions, current standard radiation dose remains inadequate for local control [18]. Other institutions reported higher gross tumor doses ranging from 63 to 70 Gy which were well tolerated even with the 3D-CRT technique [5-7,19]. Semrau et al. [20] reported the acute toxicity and long-term outcome of 15 elderly esophageal cancer patients (>70) with multiple co-morbidities who were treated with concurrent chemoradiation to a gross tumor dose of 63 Gy. Radiation treatment was well tolerated and there was no difference in survival compared to younger patients [20]. However, 3D-RT technique was associated with significant long-term toxicities because of excessive radiation dose to the lungs and hearts, resulting in pneumonitis and heart failures and/or cardiac arrythmia. Death may ensue in long-term cancer survivors from cardiac or pulmonary complications [19,21-23]. Elderly patients (>75) may be at significant risk for cardiac complications compared to younger patients because of the pre-existing co-morbidities such as heart disease [23]. Thus, it is imperative for the clinicians to reduce excessive cardiac and lungs irradiation with newer radiation modalities. Intensity-modulated radiotherapy can significantly spare the lungs from irradiation. La et al. [24] reported no grade 3-4 pneumonitis in 30 patients with locally advanced esophageal cancer who underwent pre-operative concurrent chemoradiation. Tomotherapy-based IGRT by virtue of its steep dose gradient and daily CT imaging allowing for reduced PTV margins may significantly decrease radiation dose to normal tissues and improve tolerance to chemoradiation in elderly cancer patients [25,26]. Nguyen et al. [13] reported significant reduction of cardiac and lungs irradiation in a dosimetric study comparing tomotherapy to 3D-CRT in patients with distal esophageal cancers. Indeed, we did not observe any grade 3-4 cardiac or pulmonary complications in our study despite the fact that most of the patients had multiple co-morbidity factors that preclude surgery and a higher gross tumor dose. Thus, our study corroborated the lack of serious cardiac or lungs injury with IMRT or IGRT [11,14,24]. Indeed, a review of the literature in esophageal cancer studies where a higher dose of radiation was delivered to the GTV similar to our study but with the conventional 3D-CRT technique revealed a higher rate of grade 3-4 complications. Liu et al. [27] reported the late toxicity of 111 patients with locally advanced esophageal cancer randomized to radiotherapy alone (n = 57) or concurrent chemoradiation (n = 54). A dose of 41.4 Gy in 1.8 Gy/fraction was delivered to the CTV followed by a boost of 27 Gy in 1.5 Gy twice to the GTV (total GTV dose = 68.4 Gy). Grade 3-4 toxicities occurred in 32 patients (29%) (pulmonary fibrosis: 21, esophageal stenosis: 10, pericarditis: 1). Five patients died from the pulmonary fibrosis. Toita et al. [28] treated 30 patients stage I-III esophageal carcinoma with concurrent chemoradiation. The CTV was treated to 39.6 Gy in 1.8 Gy/fraction followed by a GTV boost to achieve a total GTV dose of 66.6 Gy. There was no death but 23 patients (77%) developed grade 3-4 toxicities mainly hematologic and gastrointestinal. Five patients developed deterioration of their pulmonary function following treatment. Huzmulu et al. [29] also corroborated the high rate of grade 3-4 toxicities with radiation dose escalation using the conventional radiotherapy technique. 46 patients with stage II-III esophageal cancer were treated with chemoradiation to a total GTV dose of 66 Gy in 2 Gy/fraction. One patient died from neutropenic septicemia. 87.5% of the patients developed grade 3-4 toxicities. Thus, because of the large volume of normal tissues irradiated to a higher dose of radiation, toxicity of the treatment remains the limiting factor of radiation dose escalation with 3D-CRT. Welsh et al. [30] demonstrated in a dosimetric study that radiation dose escalation was feasible with IMRT for esophageal cancer because of the sparing of normal organs compared to 3D-CRT. Preliminary study of IMRT for radiation dose escalation to 68.1 Gy to the GTV for patients with esophageal cancer is promising. Only one out 20 patients developed grade 3 toxicity following chemoradiation [31]. The low toxicity of IMRT for radiation dose escalation in patients with esophageal cancer corroborated our results and should be investigated in the future.

Despite the small number of patients, the study shows the feasibility of implementing advanced concepts of radiotherapy, notably integrating PET-CT diagnostic imaging and chemotherapy. In a recent review, Fokas et al. [32] illustrate the potential of IGRT and PET-CT-based radiotherapy planning to further improve the therapeutic ratio of concurrent chemoradiation for esophageal cancer. Our study illustrates the validity of this concept that needs to be corroborated in future prospective studies.

The limitations of our study include the heterogeneity of our patients (widely different doses and fractionation; definitive and postoperative chemoradiotherapy; various anatomic tumor locations; different tumor histologies), small number of patients and lack of GTV volume information. However, the heterogeneity reflects a real life situation as advanced esophageal cancer covers a wide range of different patients and tumor biology. Beyond the scope of our study, the key issue in future trials will be to address what should be the major stratifying factors that would need to be taken into account.

## Conclusion

Intensity-modulated and image-guided radiotherapy may provide curative dose of radiation in patients with locally advanced esophageal cancer with acceptable complications despite pre-existing co-morbidities. Prospective studies with a large number of patients should be performed to assess the effectiveness of these new radiotherapy techniques to improve loco-regional control and patient quality of life.

## Competing interests

On behalf of all authors, the corresponding author states the following: the authors have no conflict of interest.

## Authors’ contributions

NPN, SJ, and LS collected the data. All authors participated in the study design, data interpretation, and writing of draft. All authors read and approve the manuscript.

## Pre-publication history

The pre-publication history for this paper can be accessed here:

http://www.biomedcentral.com/1471-2407/14/265/prepub
